# Evaluation of the Effects of Wax Films Formulated With Thyme and Laurel Essential Oils on Chemical and Microbiological Quality Characteristics of Rainbow Trout Fillets During Storage

**DOI:** 10.1002/fsn3.71950

**Published:** 2026-06-10

**Authors:** Ayşe Janseli Denizkara, Gökhan Akarca, Ramazan Şevik, Çiğdem Aşçioğlu, Senem Güner

**Affiliations:** ^1^ Faculty of Engineering, Department of Food Engineering Afyon Kocatepe University Afyonkarahisar Türkiye

**Keywords:** beeswax, essential oil, polyunsaturated fatty acid, rainbow trout

## Abstract

This study investigated the effects of beeswax coatings, with and without added thyme and bay laurel essential oils, on the quality of trout fillets during storage. Throughout storage, pH, a_w_, TBARS, TVB‐N, hardness, gumminess, chewiness, resilience, L*, and a* values increased, while adhesiveness, springiness, cohesiveness, and b* values decreased. Saturated fatty acids increased in all samples, while monounsaturated fatty acids decreased. Polyunsaturated fatty acids rose in coated samples but declined in the control. The addition of essential oils significantly enhanced preservation, with stronger effects at higher concentrations. On the final storage day, trout fillets coated with 4% laurel essential oil had the lowest TBARS (0.020 mgMA/kg) and TVB‐N (7.05 mg/100 g), as well as the best textural properties. Coatings with thyme oil maintained the highest monounsaturated fatty acids, while laurel oil preserved the most polyunsaturated fats. Overall, essential oil‐enriched beeswax coatings improved freshness, texture, and nutritional quality while reducing spoilage.

## Graphical abstract

This graphical abstract illustrates the superior preservative effects of essential oil‐enriched beeswax coatings on rainbow trout fillets compared to conventional methods. The application of these natural active coatings, particularly with 4% laurel essential oil, significantly slowed biochemical spoilage, improved textural attributes, and preserved polyunsaturated fatty acids. These findings highlight a sustainable, effective approach for extending the shelf life and nutritional value of stored trout products.

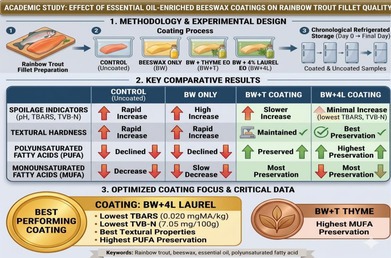

## 1 Background

Rainbow trout ( *Oncorhynchus mykiss* ), a member of the Salmonidae family native to the Pacific coasts of North America and Russia, is widespread in regions with cool water temperatures worldwide (Behnke 1992). Rainbow trout is among the main farmed fish species, accounting for 97% of the total production volume (D'Agaro et al. 2022). Fish spoilage during storage involves complex interactions between microbial growth, chemical changes, and physical properties. The primary cause of fish spoilage is microbial contamination, which leads to undesirable biochemical transformations (Kaszab et al. 2022). In addition to microbial activity, lipid hydrolysis and oxidation lead to a decrease in the quality of stored fish and negatively impact their nutritional value (Rathod et al. 2021). Additionally, biogenic amines formed during storage also contribute to the deterioration of product quality (Park et al. 2010).

Beeswax, one of the important bee products, is a complex mixture of homologous neutral lipids containing alkanes, alkenes, free fatty acids, monoesters, diesters, hydroxyesters, etc., whose physical and chemical properties vary depending on the bee species from which it is produced (Ochoa et al. 2017). Beeswax is generally recognized as safe (GRAS) by food authorities, allowing for broader use in various food formulations (Zhang et al. 2024). Used as a coating material in many different areas of the food industry for many years, beeswax is used primarily for purposes such as product protection, preventing/delaying the growth of aerobic microorganisms on surfaces to prevent oxidation, ensuring product ripening during storage, and extending the shelf life of food (Topal et al. 2020). Essential oils, thanks to the phenolic compounds they contain, not only protect foods against spoilage and disease thanks to their antioxidant and antimicrobial effects but also meet the growing consumer demand for clean‐label products (Rodilla et al. 2024; Camargo et al. 2019). The use of essential oil‐infused beeswax in food applications offers numerous benefits, including enhanced oxidative stability, improved antimicrobial properties, and attractive sensory properties. This not only supports the development of safer and higher‐quality food products but also aligns with current consumer demands for natural, clean‐label ingredients (Hromiš et al. 2017).

Recent pioneering studies have highlighted the potential of essential oil‐enriched wax films as highly effective active packaging solutions. These formulations not only provide a robust physical barrier against moisture loss and oxygen penetration but also exhibit sustained antimicrobial and antioxidant activities, significantly prolonging the shelf life of highly perishable foods like fish fillets (Hromiš et al. 2017; Öğütcü et al. 2015).

This study aimed to determine the changes in the quality of trout fillets coated with beeswax and a mixture obtained by adding essential oils (thyme and bay) to the beeswax during storage. In this context, it was aimed to reveal the physical, chemical, and microbiological properties of fish fillets coated with a wax mixture obtained by adding different proportions of thyme and laurel essential oils, as well as the differences in thiobarbituric acid (TBA) and total volatile basic nitrogen (TVB‐N) values and fatty acid profiles that occur during the storage process.

## 2 Methods

### 2.1 Study Design and Participants

The trout ( *Oncorhynchus mykiss* ) used in this study were purchased post‐mortem as gutted and cleaned fish from a commercial production facility operating in Afyonkarahisar province. The beeswax and fixed oils (thyme and bay laurel) used in coating the fish were supplied by a company operating in the Akşehir district (Uğurluoğlu Tic. A.Ş., Konya, Turkey).

### 2.2 Coating the Fish

After gutting and cleaning the fish for this study, we appropriately filleted them. The beeswax was first cut into small pieces using a knife and placed in a suitably sized conical flask. The beeswax was then placed in a container containing water at 90°C. Once the beeswax had completely melted, impurities collected on the surface were removed. Thyme and laurel essential oils were added in the amounts found from earlier tests (2% and 4%). The raw, cold fillets were dipped into the mixture for 3–5 s. The molten wax rapidly solidified upon contact with the cold fillet surface, instantly forming a protective film without causing heat shock or raising the internal temperature of the underlying muscle tissue. The coating process was carried out separately, twice. The coated fillets were stored in aerobic lidded plastic storage containers in a refrigerator at 0°C–4°C for 10 days.

### 2.3 Physicochemical Analyses

#### 2.3.1 pH and Water Activity (a_w_) Values

pH values of trout samples were determined using a calibrated pH meter (ST 5000, USA) according to AOAC (2016). The water activity values of the samples were determined using a water activity tester (Novasina Lab Touch‐Aw Lachen, Switzerland), according to AOAC (2016).

#### 2.3.2 Thiobarbituric Acid (TBA) and Total Volatile Basic Nitrogen (TVB‐N) Values

TBA values of the samples during storage were determined according to the methods specified by Tarladgi et al. (1960), and TVB‐N values were determined according to the methods specified by İnal (1992).

#### 2.3.3 Color Values

Color analysis of the samples was carried out by making three parallel measurements using a Konica Minolta (Chromameter CR‐400) device (Babikova et al. 2020).

#### 2.3.4 Texture Profile Analysis (TPA) Values

TPA values of fish samples were determined at room temperature using a texture analyzer (TA‐XT2i; Stable Microsystems Ltd., Surrey, England) with a 5 kg load cell. Measurements were taken using a cylindrical aluminum probe (P/50, 50 mm diameter, Stable Micro Systems LTD, Godalming, England). Before analysis, fish fillets were divided into 10 × 10 mm portions. Pre‐test, test, and post‐test speeds were set as 1, 5, and 5 mm/s, respectively. The type of deformation applied to the samples during the analysis was selected as strain and set to 40%. Measurements were made three times to determine the TPA profile of each sample (Rampelotto et al. 2021).

#### 2.3.5 Microbiological Analyses

10 g of fish samples were taken under sterile conditions and transferred to sterile stomacher bags (Spa‐174,538, Lp Italiana, Milano, Italy), and serial dilutions up to 10^−4^ were prepared, and analyses were performed using the spread plate method from these dilutions. Analysis of total aerobic mesophilic bacteria (TAMB) and total psychrophilic bacteria (TAPB) counts was carried out using Plate Count Agar (PCA) (Merck, Germany, 1.05463). The cultured petri dishes were incubated under aerobic conditions at 30°C in an incubator (MM Incucell 55, Germany) for 48–72 h for TAMB count and at 4°C in the refrigerator for 5–7 days for TAPB count (ISO 2013a, 2013b; Halkman and Sağdaş 2011). For the analysis of total yeast/mold (TMB) counts, Potato Dextrose Agar (PDA Merck 1.10130, Germany) was used, adjusted to pH 3.5 using 10*%* tartaric acid, and the cultured petri dishes were incubated in an incubator (MMM Incucell 55, Germany) at 22°C under aerobic conditions for 5–7 days (ISO 2008). For the enumeration of lactic acid bacteria, Man Rogosa and Sharpe Agar (MRS, Merck 1.10660, Germany) was used. The cultured petri dishes were placed in jars (Merck 1.16387, Germany) and incubated in an incubator (Daihan, IG50, Malaysia) at 30°C under anaerobic conditions for 24–48 h (Kneifel and Berger 1994). For the analysis of total coliform group bacteria (TCGB) counts, Violet Red Bile Agar (VRB, Merck 1.01406, Germany) was used. Petri dishes were incubated in an incubator (MMM Incucell 55, Germany) under aerobic conditions at 30°C for 24–48 h, then dark red colonies were counted (ISO 1991). For the counts of lipolytic bacteria, Tributyrin Agar (M157, Himedia, India) was used. The cultured petri dishes were incubated in an incubator (MMM Incucell 55, Germany) under aerobic conditions at 30°C for 48–72 h (Halkman 2005).

#### 2.3.6 Determination of Fatty Acid Composition

Fatty acid analyses of trout samples were carried out using a GC/MS (AGILENT 5975 C AGILENT 7890A GC) analyzer. The MSDCHEM program and a DB WAX (50*0.20 mm, 0.20 μm) column were used in the device. The initial temperature of the oven was determined to be 80°C; after 4 min, the temperature was increased to 175°C with an increase of 13°C per minute. It was held at this temperature for 27 min. Then, the temperature reached 215°C with an increase of 4°C per minute and was held at this temperature for 5 min. Then, the temperature reached 240°C with an increase of 4°C per minute and was held for 15 min. The detector and injector temperature was 240°C. The injection volume was set as 1 μL, and the detector and injector temperatures were set as 240°C. In the analyses, HCl was used as a derivatizer at a concentration of 1.5 M, the derivatization temperature was 80°C, and the derivatization time was 2 h (Bardakçı and Secilmis 2006).

#### 2.3.7 Statistical Analysis

Statistical values were calculated using the SPSS V 23.0.0 (SPSS Inc., Chicago, IL, USA) statistical package program. Data obtained from the analyses were evaluated using analysis of variance. The significance level was determined using the Duncan test (*p* < 0.05), and the impact of the results was determined by calculating the Pearson correlation coefficient.

## 3 Results

The type of sample, how long it was stored, and the combination of sample type and storage time (not including TBARS values) significantly affected the pH, a_w_, TBARS, and TVB‐N values of the samples (*p* < 0.0001). Additionally, the interaction of sample type negatively impacted pH, TBARS, and TVB‐N values, while it positively affected a_w_ (Table 1). The control sample showed the largest increase in pH during storage, with a 1.33 increase, while samples coated with beeswax containing 4% thyme oil increased by 0.01.

**TABLE 1:** Physicochemical analysis results of samples.

Analysis	Samples	Storage time (Day)			
		1.	4.	7.	10.
pH	Control	6.41 ± 0.01^Ca^	6.20 ± 0.01^Cb^	7.12 ± 0.02^Ba^	7.74 ± 0.03^Aa^
	Beewax	6.38 ± 0.01^Dab^	6.26 ± 0.01^Ca^	6.51 ± 0.02^Bb^	6.72 ± 0.01^Ab^
	Thyme %2	6.32 ± 0.01^Acd^	6.21 ± 0.01^Cb^	6.27 ± 0.01^Bbc^	6.32 ± 0.01^Acd^
	Thyme %4	6.29 ± 0.01^Ae^	6.21 ± 0.01^Bb^	6.24 ± 0.01^Bc^	6.30 ± 0.01^Ad^
	Laurel %2	6.35 ± 0.01^Abc^	6.24 ± 0.01^Bab^	6.34 ± 0.01^Abc^	6.36 ± 0.01^Ac^
	Laurel %4	6.31 ± 0.01^Ad^	6.21 ± 0.02^Bb^	6.30 ± 0.01^Abc^	6.34 ± 0.01^Ac^
	**Interactions**	**p‐value**		**r**	
	Sample (S)	< 0.0001		−0.481**	
	Storage Time (ST)	< 0.0001		0.359*	
	S × ST	< 0.0001		—	
a_w_	Control	0.961 ± 0.001^Aa^	0.909 ± 0.001^Bb^	0.876 ± 0.001^Cd^	0.795 ± 0.002^De^
	Beewax	0.959 ± 0.001^Aa^	0.950 ± 0.001^Ba^	0.927 ± 0.001^Cc^	0.906 ± 0.001^Dd^
	Thyme %2	0.960 ± 0.001^Aa^	0.953 ± 0.002^Ba^	0.939 ± 0.001^Cb^	0.915 ± 0.001^Dc^
	Thyme %4	0.959 ± 0.001^Aa^	0.952 ± 0.001^Ba^	0.943 ± 0.002^Ca^	0.920 ± 0.001^Db^
	Laurel %2	0.960 ± 0.001^Aa^	0.952 ± 0.001^Ba^	0.943 ± 0.001^Ca^	0.921 ± 0.001^Db^
	Laurel %4	0.960 ± 0.001^Aa^	0.951 ± 0.001^Ba^	0.946 ± 0.001^Ca^	0.925 ± 0.001^Da^
	**Interactions**	**p‐value**		**r**	
	Sample (S)	< 0.0001		0.445**	
	Storage Time (ST)	< 0.0001		−0.638**	
	S × ST	< 0.0001		—	
TBARS (mg MA/kg)	Control	0.036 ± 0.01^Ca^	0.041 ± 0.03^BCa^	0.045 ± 0.02^Ba^	0.052 ± 0.02^Aa^
	Beewax	0.025 ± 0.02^Bb^	0.028 ± 0.01^Bb^	0.029 ± 0.02^Bb^	0.037 ± 0.02^Ab^
	Thyme %2	0.020 ± 0.01^Bc^	0.021 ± 0.01^Bc^	0.024 ± 0.01^ABc^	0.026 ± 0.01^Ac^
	Thyme %4	0.017 ± 0.01^Bcd^	0.018 ± 0.02^Bcd^	0.021 ± 0.01^Bcd^	0.026 ± 0.02^Ac^
	Laurel %2	0.015 ± 0.05^Ccd^	0.018 ± 0.01^BCcd^	0.020 ± 0.01^ABcd^	0.023 ± 0.01^Acd^
	Laurel %4	0.013 ± 0.06^Bd^	0.015 ± 0.01^ABd^	0.018 ± 0.01^ABd^	0.020 ± 0.01^Ad^
	**Interactions**	**p‐value**		**r**	
	Sample (S)	< 0.0001		−0.824**	
	Storage Time (ST)	< 0.0001		0.359*	
	S × ST	0.098		—	
TVB‐N (mg/100 g)	Control	9.68 ± 0.04^Da^	14.37 ± 0.06^Ca^	27.81 ± 0.19^Ba^	31.47 ± 0.04^Aa^
	Beewax	8.11 ± 0.03^Db^	9.79 ± 0.02^Cb^	11.41 ± 0.03^Bb^	12.91 ± 0.03^Ab^
	Thyme %2	7.66 ± 0.06^Dc^	7.95 ± 0.02^Cc^	8.23 ± 0.03^Bc^	8.95 ± 0.04^Ac^
	Thyme %4	7.09 ± 0.03^Cd^	7.20 ± 0.04^Cd^	7.39 ± 0.07^Bd^	7.59 ± 0.05^Ad^
	Laurel %2	7.23 ± 0.02^Ce^	7.32 ± 0.02^BCe^	7.40 ± 0.03^ABd^	7.48 ± 0.06^Ae^
	Laurel %4	6.86 ± 0.03^Cf^	6.93 ± 0.04^Cf^	7.01 ± 0.03^ABe^	7.05 ± 0.05^Af^
	**Interactions**	**p‐value**		**r**	
	Sample (S)	< 0.0001		−0.630**	
	Storage Time (ST)	< 0.0001		0.308*	
	S × ST	< 0.0001		—	

*Note:* a–f (↓): Values with the same capital letters in the same column for each analysis differ significantly (*p* < 0.05). A (→) D: Values with the same capital letters in the same column for each analysis differ significantly (*p* < 0.05) *p* < 0.0001: Highly significant.

* Correlation is significant at the 0.05 level (2‐tailed).

** Correlation is significant at the 0.01 level (2‐tailed).

During storage, fish meat undergoes biochemical changes such as autolysis and muscle protein breakdown, leading to changes in the internal environment that can increase pH levels. Specifically, the activity of metabolism and the breakdown of proteins, which release basic amines, help cause this increase (Malik et al. 2021). Additionally, microbial growth can influence pH increases during storage (Sarika et al. 2019). Storage‐related pH increases were less pronounced in beeswax‐coated samples compared to control samples, and the addition of essential oils to the beeswax further reduced the pH increase. This result stems from the beeswax slowing oxidative spoilage and the added essential oils affecting microbial growth.

Similar to our results, Erkan et al. (2011) investigated the effects of thyme and bay laurel essential oils on *Pomatomus saltatrix* fillets. They found that pH decreased from 6.68 in the control group to 6.45 in thyme oil‐treated samples, indicating that thyme oil has effective antimicrobial properties in preserving fish quality. Similarly, Yıldız (2014) reported that thyme essential oil reduced the pH of smoked rainbow grapes, and this decrease in pH was due to the thyme oil's phenolic compounds, which enhance its preservative properties.

The a_w_ values of the samples decreased during storage (*p* < 0.05). This decrease during storage was highly significant in the control sample, while the decrease during storage in the beeswax‐coated samples was less significant. Furthermore, the addition of oil to the coating material slowed the rate of a_w_ decrease during storage. The rate of a_w_ decrease also slowed down in parallel with the amount of oil added (*p* < 0.05).

Among all the samples tested, the laurel sample with 4*%* oil exhibited the smallest change in a_w_ value (0.035) during storage. The samples' a_w_ values dropped during storage as a result of surface water loss. In general, the addition of hydrophobic materials such as beeswax to edible film formulations has shown positive results in coating fresh produce in terms of barrier properties, mechanical properties, and moisture control, as well as maintaining quality and safety (Ochoa et al. 2017). The beeswax coating reduced water loss during storage, and the addition of oil to the beeswax further reduced it.

Lipid oxidation is a significant concern in fish storage, leading to off‐flavors, nutrient losses, and quality deterioration (Kuroda et al. 2020). Fish samples showed an increase (*p <* 0.05) during storage. The control sample showed the largest increase in TBARS values during storage, with an increase of 0.0165 mg MA/kg. The TBARS values in beeswax‐coated samples increased less during storage compared to the control sample, while this increase was much less in samples containing essential oils (Table 1).

The beeswax coating creates a protective barrier that limits oxygen exposure; this prevents oxygen from being the primary trigger of lipid oxidation, which leads to spoilage in fish (Khan et al. 2015).

Research shows that thyme and bay laurel essential oils possess significant antioxidant activity thanks to their rich polyphenolic compounds. Studies have shown that thyme and bay laurel essential oils can inhibit lipid peroxidation (Durmuş et al. 2023; Mahale and Gattani 2023). These results explain why the addition of essential oils to beeswax further reduced the TBARS value in our study.

Total Volatile Basic Nitrogen (TVB‐N) is an important chemical indicator used to assess the freshness and quality of fish products. This value primarily reflects accumulated nitrogenous compounds resulting from spoilage processes, including protein degradation by microbial and enzymatic activity, generating trimethylamine (TMA), dimethylamine (DMA), ammonia, and other similar nitrogen compounds. Consequently, the TVB‐N values of the trout samples significantly increased during the storage period (*p* < 0.05). The TVB‐N value was highest on the last day of storage.

The control sample contained 31.47 mg/100 g of TVB‐N, while the lowest TVB‐N value was found in the Laurel 4% sample at 7.05 mg/100 g.

Research shows that the application of wax coatings can significantly affect biochemical changes during seafood storage, particularly by reducing TVB‐N levels. Specifically, wax coatings create a barrier that limits oxygen exposure, delaying microbial growth and biochemical reactions that result in increased TVB‐N levels (Xu et al. 2017; Lan et al. 2022).

Additionally, the addition of essential oils to the coatings has been reported to have even more beneficial effects in controlling spoilage. Consistent with our research results, Xu et al. (2017) reported that the use of garlic and ginger essential oil coatings on fish fillets resulted in a significant decrease in TVB‐N values due to the coatings' antimicrobial and antioxidant properties that minimize spoilage. Yıldız (2014) reported that thyme essential oil minimized the formation of potential spoilage indicators such as volatile basic nitrogen (TVB‐N) in smoked rainbow trout.

The sample type, storage time, and sample type × storage time interactions were significantly effective (*p* < 0.0001) on all textural values of the trout samples. Furthermore, the sample type interaction also showed a significant negative correlative effect on all textural values. The storage time interaction revealed a significant negative correlative effect on adhesiveness, springiness, and cohesiveness values and a significant positive correlative effect on resilience values (Table 2).

**TABLE 2:** TPA analysis results of samples.

Analysis	Samples	Storage time (Day)			
		1.	4.	7.	10.
Hardness (N)	Control	679.27 ± 5.56^Da^	774.94 ± 6.49^Ca^	918.51 ± 21.06^Ba^	1138.29 ± 4.99^Aa^
	Beewax	584.45 ± 3.94^Db^	628.07 ± 6.13^Cb^	691.58 ± 2.77^Bb^	768.21 ± 4.23^Ab^
	Thyme %2	510.38 ± 2.93^Cc^	547.40 ± 5.44^Bc^	580.75 ± 3.53^Ac^	591.69 ± 5.02^Ac^
	Thyme %4	360.01 ± 9.70^Cd^	385.07 ± 4.86^Bd^	393.85 ± 2.24^ABd^	403.77 ± 3.62^Ad^
	Laurel %2	471.82 ± 7.65^Ce^	489.33 ± 5.93^Be^	500.41 ± 1.48^Be^	519.42 ± 6.05^Ae^
	Laurel %4	321.82 ± 4.09^Cf^	340.89 ± 6.24^Bf^	353.24 ± 3.77^Bf^	369.90 ± 3.16^Af^
	*Interactions*	*p‐value*		*r*	
	Sample (S)	< 0.0001		−0.835**	
	Storage Time (ST)	< 0.0001		0.273	
	S × ST	< 0.0001		—	
Adhesiveness (g. s)	Control	−8.54 ± 0.47^Aa^	−9.60 ± 0.24^Ba^	−10.42 ± 0.34^Ba^	−12.67 ± 0.16^Ca^
	Beewax	−13.82 ± 0.35^Ab^	−15.67 ± 0.49^Bb^	−18.89 ± 0.33^Cb^	−21.61 ± 0.40^Db^
	Thyme %2	−17.74 ± 0.11^Ac^	−21.28 ± 0.32^Bc^	−24.71 ± 0.23^Cc^	−29.93 ± 0.21^Dc^
	Thyme %4	−19.44 ± 0.33^Ad^	−23.99 ± 0.12^Bd^	−29.4 ± 0.39^Cd^	−34.63 ± 0.11^Dd^
	Laurel %2	−18.82 ± 0.55^Ad^	−23.27 ± 0.19^Be^	−28.02 ± 0.20^Ce^	−32.46 ± 0.44^De^
	Laurel %4	−20.46 ± 0.33^Ae^	−25.91 ± 0.18^B^f	−31.42 ± 0.30^Cf^	−36.15 ± 0.08^Df^
	*Interactions*	*p‐value*		*r*	
	Sample (S)	< 0.0001		−0.745**	
	Storage Time (ST)	< 0.0001		−0.549**	
	S × ST	< 0.0001		—	
Springiness	Control	0.824 ± 0.001^Aa^	0.785 ± 0.001^Ba^	0.699 ± 0.005^Ca^	0.661 ± 0.001^Da^
	Beewax	0.786 ± 0.001^Ab^	0.724 ± 0.002^Bb^	0.658 ± 0.004^Cb^	0.595 ± 0.001^Db^
	Thyme %2	0.626 ± 0.004^Ac^	0.589 ± 0.005^Bc^	0.461 ± 0.006^Cc^	0.380 ± 0.008^Dc^
	Thyme %4	0.537 ± 0.005^Ae^	0.479 ± 0.004^Be^	0.400 ± 0.008^Cd^	0.281 ± 0.010^De^
	Laurel %2	0.607 ± 0.006^Ad^	0.578 ± 0.004^Bd^	0.450 ± 0.003^Cc^	0.336 ± 0.006^Dd^
	Laurel %4	0.514 ± 0.002^Af^	0.463 ± 0.003^Bf^	0.387 ± 0.005^Cd^	0.248 ± 0.011^Df^
	*Interactions*	*p‐value*		*r*	
	Sample (S)	< 0.0001		−0.738**	
	Storage Time (ST)	< 0.0001		−0.562**	
	S × ST	< 0.0001		—	
Cohesiveness	Control	0.627 ± 0.009^Aa^	0.610 ± 0.004^Ba^	0.591 ± 0.004^Ca^	0.561 ± 0.004^Da^
	Beewax	0.607 ± 0.004^Ab^	0.592 ± 0.002^Bb^	0.567 ± 0.004^Cb^	0.549 ± 0.002^Db^
	Thyme %2	0.590 ± 0.003^Ac^	0.569 ± 0.004^Bc^	0.561 ± 0.001^Cb^	0.540 ± 0.003^Dc^
	Thyme %4	0.565 ± 0.004^Ade^	0.540 ± 0.004^Be^	0.527 ± 0.004^Cd^	0.514 ± 0.004^De^
	Laurel %2	0.576 ± 0.004^Ad^	0.560 ± 0.001^Bd^	0.548 ± 0.002^Cc^	0.529 ± 0.001^Dd^
	Laurel %4	0.553 ± 0.003^Ae^	0.529 ± 0.003^Bf^	0.517 ± 0.005^Ce^	0.506 ± 0.004^Df^
	*Interactions*	*p‐value*		*r*	
	Sample (S)	< 0.0001		−0.715**	
	Storage Time (ST)	< 0.0001		−0.620**	
	S × ST	< 0.0001		—	
Gumminess (N)	Control	426.27 ± 9.73^Da^	473.11 ± 6.70^Ca^	542.88 ± 16.34^Ba^	638.59 ± 7.63^Aa^
	Beewax	354.75 ± 0.88^Db^	372.12 ± 2.30^Cb^	392.47 ± 4.02^Bb^	422.13 ± 3.95^Ab^
	Thyme %2	301.12 ± 0.28^Cc^	311.75 ± 5.03^Bc^	325.79 ± 1.16^Ac^	319.50 ± 1.04^Ac^
	Thyme %4	203.42 ± 7.01^Ae^	208.13 ± 3.99^Ae^	207.55 ± 0.49^Ae^	207.73 ± 0.44^Ae^
	Laurel %2	272.02 ± 6.08^Ad^	274.03 ± 4.01^Ad^	274.47 ± 0.25^Ad^	275.03 ± 3.57^Ad^
	Laurel %4	177.97 ± 3.17^Bf^	180.32 ± 2.33^Bf^	182.79 ± 0.21^ABf^	187.34 ± 0.30^Af^
	*Interactions*	*p‐value*		*r*	
	Sample (S)	< 0.0001		−0.873**	
	Storage Time (ST)	< 0.0001		0.164	
	S × ST	< 0.0001		—	
Chewiness (N)	Control	351.25 ± 8.62^Ca^	371.38 ± 4.59^Ba^	379.70 ± 8.74^Ba^	422.11 ± 5.94^Aa^
	Beewax	278.83 ± 0.57^Ab^	269.60 ± 0.87^Bb^	258.44 ± 1.26^Cb^	251.16 ± 1.75^Db^
	Thyme %2	188.65 ± 1.31^Ac^	183.76 ± 1.42^Bc^	150.35 ± 1.53^Cc^	121.40 ± 2.31^Dc^
	Thyme %4	109.36 ± 4.77^Ae^	99.80 ± 2.64^Be^	83.02 ± 1.95^Ce^	58.37 ± 2.17^De^
	Laurel %2	165.13 ± 5.23^Ad^	158.38 ± 1.15^Ad^	123.51 ± 0.66^Bd^	92.56 ± 2.95^Cd^
	Laurel %4	91.57 ± 2.01^Af^	83.48 ± 0.57^Bf^	70.83 ± 0.98^Cf^	46.55 ± 1.91^Df^
	*Interactions*	*p‐value*		*r*	
	Sample (S)	< 0.0001		−0.886**	
	Storage Time (ST)	< 0.0001		−0.115	
	S × ST	< 0.0001		—	
Resilience	Control	0.268 ± 0.004^Da^	0.368 ± 0.005^Ca^	0.446 ± 0.006^Ba^	0.508 ± 0.006^Aa^
	Beewax	0.219 ± 0.002^Db^	0.313 ± 0.006^Cb^	0.357 ± 0.012^BCb^	0.417 ± 0.008^Ab^
	Thyme %2	0.215 ± 0.004^Cb^c	0.282 ± 0.008^Bc^	0.300 ± 0.006^Bc^	0.394 ± 0.011^Ac^
	Thyme %4	0.199 ± 0.004^Dd^	0.242 ± 0.003^Cd^	0.266 ± 0.006^Bd^	0.303 ± 0.006^Ae^
	Laurel %2	0.207 ± 0.001^Dcd^	0.270 ± 0.004^Cc^	0.290 ± 0.009^Bc^	0.369 ± 0.008^Ad^
	Laurel %4	0.188 ± 0.004^De^	0.231 ± 0.005^Ce^	0.257 ± 0.004^Be^	0.278 ± 0.004^Af^
	*Interactions*	*p‐value*		*r*	
	Sample (S)	< 0.0001		−0.578**	
	Storage Time (ST)	< 0.0001		0.716**	
	S × ST	< 0.0001		—	

*Note:* a–f (↓): Values with the same capital letters in the same column for each analysis differ significantly (*p* < 0.05). A (→) D: Values with the same capital letters in the same column for each analysis differ significantly (*p* < 0.05) *p* < 0.0001: Highly significant.

** Correlation is significant at the 0.01 level (2‐tailed).

The hardness values of the samples increased during storage (*p* < 0.05). The control sample showed the greatest increase in hardness values during storage, with a value of 459.01 N, while the thyme 4% sample showed the least increase, with a value of 43.75 N. The wax coating accelerated the decrease in hardness values, and the addition of essential oil to the wax further enhanced this effect.

Post‐mortem biochemical changes experienced by fish can significantly affect the tissue. While fish are stored, natural enzymes and bacteria break down muscle proteins, which changes the hardness and texture of the fish. The activity of enzymes keeps going while the fish is stored, and at first, the breakdown of proteins happens slowly, but as time goes on, the fish becomes harder because the way the muscle proteins interact changes. This phenomenon can generally be associated with changes in the water‐holding capacity of fish tissues (Hu et al. 2020).

When mixed with beeswax, coatings that have essential oils provide strong protection that helps keep moisture in and reduces fat breakdown, which directly impacts hardness. Furthermore, coatings enriched with essential oils act as barriers to moisture loss and affect hardness by limiting microbial growth and oxidative reactions that can lead to tissue degradation (Hwang et al. 2018). Additionally, the natural properties of beeswax, when integrated with essential oils in a coating, can form wax films that exhibit superior stability. Increases in the concentration of essential oils in coatings can be positively correlated with improved protective effects (Öğütcü et al. 2015).

Adhesiveness, springiness, and cohesiveness values of the samples decreased during storage (*p* < 0.05). On the last day of storage, the lowest adhesiveness, springiness, and cohesiveness values were found in the laurel 4% samples at −36.15 g.s., 0.248, and 0.506, respectively, while the highest values were found in the control sample at −12.67 g.s., 0.661, and 0.561. In the same way, using beeswax coating made the adhesiveness, springiness, and cohesiveness values drop faster, and adding essential oil to the beeswax made this effect even stronger (Table 2).

During fish storage, proteolytic enzymes become increasingly active, leading to the degradation of muscle proteins. Not only do endogenous proteins cause this degradation, but storage conditions may also catalyze microbial activity. Degradation causes the connections between connective tissue and muscle proteins (myosin and actin) to weaken, which worsens the texture and results in a loss of springiness, stickiness, and firmness. Furthermore, losses in water retention capacity directly affect the general sensory properties of fish, such as adhesiveness, springiness, and cohesiveness (Sun et al. 2020). Wax coatings act as a barrier against oxygen while maintaining the moisture levels of the samples.

It helps minimize lipid oxidation, thereby reducing the toughening effect associated with lipid peroxidation (Baron et al. 2007). Endogenous proteolytic enzymes, which lead to the degradation of structural proteins in muscle tissues, also play a role in textural changes. Storage conditions, such as temperature and packaging methods, can significantly affect the activity of these enzymes (Ghorai and Dora 2021). The lower penetration of these enzymatic agents in wax‐coated fish helped maintain some flexibility for longer periods compared to their uncoated counterparts. The combination of wax coatings with essential oils has been shown to prevent moisture loss and preserve the texture of fish samples. Furthermore, wax coatings with added essential oils can cause changes in lipid and protein structures at the molecular level, potentially affecting muscle fiber elasticity and cohesion, which are essential for flexibility (Santos et al. 2019).

Gumminess and resilience values of trout samples increased during storage. Chewiness values, on the other hand, increased in the control sample but decreased in the wax‐coated samples (*p* < 0.05). The highest increases in gumminess and resilience values during storage were determined to be in the control samples, with values of 212.32 N and 0.240, respectively (Table 2). Coating the samples with wax and adding essential oil to the coating material reduced the effect of the increase in gumminess and resilience values during storage. Furthermore, wax coating caused a decrease in chewiness values compared to the control sample. Although the gumminess, resilience, and chewiness values of trout fillets changed significantly during storage, this change was significantly reduced in the wax‐coated samples. We increased this positive effect by adding essential oil to the wax. The lowest chewiness values at the end of storage were detected in laurel 4% samples with 46.55 N and thyme 4% with 58.37 N. As stored fish undergo proteolysis, the interaction between myofibrillar proteins and water alters gumminess, resilience, and chewiness. The impact of these changes increases with storage time. The main factors affecting these changes are protein/lipid oxidation and microbial activity (Zhao et al. 2012). Beeswax coating slows the degradation of structural proteins in fish fillets, preserving desirable textural qualities such as chewiness and gumminess. The addition of essential oils to the coating enhances this preservation thanks to the antioxidant and antimicrobial compounds they contain (Chakraborty et al. 2022).

Essential oils, especially those from aromatic herbs like thyme and rosemary, positively affect the quality and shelf life of fish products when incorporated into beeswax coatings. Cai et al. (2017) showed that the use of chitosan‐based coatings with essential oils could delay tissue deterioration in fish, with important parameters such as gumminess and chewiness not showing significant changes during certain storage periods. The results obtained are consistent with our research.

**FIGURE 1:**
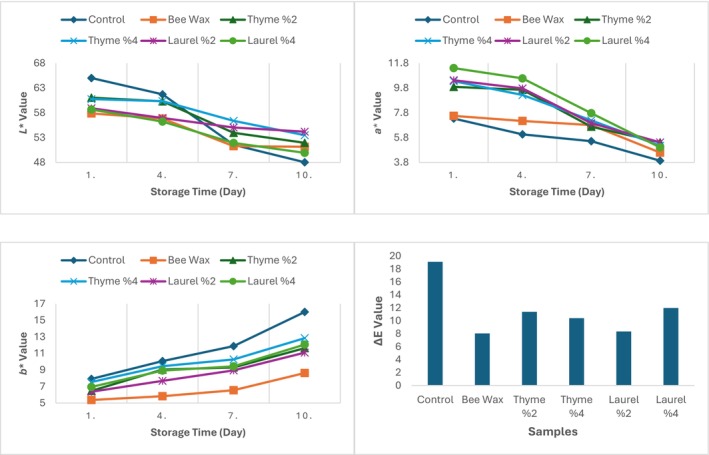
Changes in L*, a*, and b* values of trout fillets during storage. Statistical significance (*p* < 0.05) is detailed in Section 3.

The statistical evaluation (ANOVA) confirmed that the sample type, storage time, and their interactions had a highly significant impact (*p* < 0.0001) on the L*, a*, and b* values of trout samples. Significant differences between groups were determined using the Duncan test at a 95% confidence level (*p* < 0.05). While L* and a* values decreased during storage, b* values increased (*p* < 0.05). On the 10th day of storage, the lowest L* and a* values and the highest b* values were found in the control sample, with values of 48.035, 3.94, and 16.01, respectively. Conversely, the highest L* and a* values were found in the control sample, respectively (Figure 1). Coating the samples with beeswax and adding essential oil to the coating material reduced the rate of these changes.

During storage of trout fillets, the red‐green value (a*) decreases, along with the brightness value (L*). This decrease can be attributed to lipid oxidation and protein denaturation, which increase light refraction and make the meat appear whiter (Sáez et al. 2021). More specifically, oxidative degradation of carotenoids, responsible for the reddish hue in fish meat, contributes to the perceived decrease in a* values (Fellenberg 2020). On the other hand, the yellow‐blue value (b*) increases during storage and is associated with lipid oxidation processes that produce yellow pigments (Neto and Monteiro 2024). Among the samples, the control sample showed the greatest increase in ΔE during storage, with a value of 19.11, while the wax‐coated samples showed the least change, with a value of 8.03 (Figure 1).

The inclusion of essential oils in the wax coatings has been shown to reduce the rate of these changes. Essential oils from plants such as thyme, laurel, and clove have antimicrobial properties that prevent spoilage and reduce microbial growth.

These oils also contain compounds that maintain oxidation stability. This preserves the sensory properties of the fish and thus extends the time before significant changes occur in L*, a*, and b* values (Rezaeifar et al. 2020).

While myristic acid values in trout fillet samples increased during storage, palmitic, stearic, and eicosanoic acid values decreased, resulting in an increase in the total saturated fatty acid content. Similarly, the monounsaturated fatty acid levels of the samples, oleic and eicosenoic acid, decreased during storage, while palmitoleic acid increased. Conversely, the levels of polyunsaturated fatty acids decreased in the control sample during storage but increased in the other samples (Table 3). The predominant fatty acids in rainbow trout consist of palmitic acid and various PUFAs, particularly eicosapentaenoic acid (EPA) and docosahexaenoic acid (DHA). The higher presence of PUFAs during storage may increase susceptibility to oxidative stress and spoilage, thus affecting both the nutritional value and sensory properties of the fish.

**TABLE 3:** (a) Saturated fatty acids (%) of samples, (b) Monounsatured fatty acids of samples, and (c) Polyunsaturated fatty acids of samples.

Samples	Storage time (Day)	Saturated fatty acids (%)				
		Myristic acid C14:0	Palmitic acid C16:0	Stearic acid C18:0	Eikosanoic acid C20:0	∑ SFA
(a)						
Control	1.	3.850	16.907	5.37	0.33	26.457
	4.	4.165	16.885	5.31	0.27	26.63
	7.	4.415	16.778	5.26	0.20	26.653
	10.	4.550	16.615	5.21	0.18	26.555
Beewax	1.	3.895	16.893	5.42	0.32	26.528
	4.	4.305	16.866	5.33	0.27	26.771
	7.	4.590	16.778	5.24	0.22	26.828
	10.	4.710	16.772	5.16	0.19	26.832
Thyme 2%	1.	3.805	16.955	5.44	0.35	26.55
	4.	3.935	16.932	5.41	0.31	26.587
	7.	4.150	16.892	5.27	0.28	26.592
	10.	4.385	16.885	5.21	0.24	26.72
Thyme 4%	1.	3.780	17.008	5.51	0.37	26.668
	4.	3.855	16.996	5.47	0.33	26.651
	7.	3.955	16.932	5.43	0.29	26.607
	10.	4.100	16.903	5.37	0.28	26.653
Laurel %2	1.	3.780	16.978	5.49	0.35	26.598
	4.	3.905	16.965	5.44	0.33	26.64
	7.	4.085	16.935	5.32	0.30	26.64
	10.	4.180	16.903	5.26	0.29	26.633
Laurel %4	1.	3.720	17.058	5.54	0.36	26.678
	4.	3.805	17.021	5.45	0.35	26.626
	7.	3.900	16.994	5.38	0.31	26.584
	10.	4.050	16.975	5.33	0.29	26.645

Samples	Storage time (Day)	Monounsaturated fatty acids (%)			
		Palmitoleic acid C16:1	Oleic acid C18:1	Eikosenoic acid C20:1	∑ MUSFA
(b)					
Control	1.	5.01	25.21	2.57	32.79
	4.	5.02	25.15	2.55	32.72
	7.	5.00	25.12	2.56	32.68
	10.	5.02	25.06	2.55	32.63
Beewax	1.	5.02	25.33	2.58	32.93
	4.	5.02	25.29	2.57	32.88
	7.	5.03	25.27	2.55	32.85
	10.	5.02	25.26	2.56	32.84
Thyme 2%	1.	5.04	25.35	2.59	32.98
	4.	5.03	25.33	2.57	32.93
	7.	5.04	25.34	2.58	32.96
	10.	5.05	25.32	2.56	32.93
Thyme 4%	1.	5.05	25.37	2.57	32.99
	4.	5.04	25.35	2.56	32.95
	7.	5.05	25.34	2.54	32.93
	10.	5.05	25.35	2.55	32.95
Laurel 2%	1.	5.03	25.32	2.57	32.92
	4.	5.02	25.30	2.56	32.88
	7.	5.04	25.31	2.57	32.92
	10.	5.03	25.30	2.55	32.88
Laurel 4%	1.	5.04	25.33	2.55	32.92
	4.	5.03	25.32	2.54	32.89
	7.	5.03	25.30	2.53	32.86
	10.	5.04	25.31	2.54	32.89

Samples	Storage time (Day)	Pollyunsaturated fatty acids (%)					
		Linoleic acid C18:2	Linolenic acid C18:3	Arashidonic acid C20:4	Eikozapentaenoik acid C20:5	Dokosahexaenoik acid C22:6	∑PUFA
(c)							
Control	1.	13.44	0.19	1.44	1.88	5.44	22.39
	4.	13.41	0.16	1.40	1.81	5.41	22.19
	7.	13.38	0.14	1.39	1.74	5.38	22.03
	10.	13.35	0.11	1.36	1.68	5.43	21.93
Beewax	1.	13.42	0.17	1.46	1.86	5.43	22.34
	4.	13.41	0.16	1.44	1.88	5.42	22.31
	7.	13.44	0.18	1.48	1.88	5.44	22.42
	10.	13.46	0.19	1.48	1.89	5.42	22.44
Thyme 2%	1.	13.43	0.17	1.45	1.87	5.43	22.35
	4.	13.44	0.16	1.45	1.87	5.45	22.37
	7.	13.44	0.19	1.47	1.89	5.45	22.44
	10.	13.46	0.19	1.49	1.91	5.43	22.48
Thyme 4%	1.	13.44	0.20	1.46	1.86	5.43	22.39
	4.	13.45	0.21	1.47	1.85	5.45	22.43
	7.	13.42	0.19	1.49	1.88	5.46	22.44
	10.	13.36	0.23	1.50	1.92	5.48	22.49
Laurel 2%	1.	13.41	0.19	1.45	1.86	5.43	22.34
	4.	13.40	0.18	1.43	1.87	5.42	22.3
	7.	13.39	0.19	1.46	1.88	5.44	22.36
	10.	13.42	0.20	1.46	1.88	5.44	22.4
Laurel 4%	1.	13.43	0.19	1.47	1.87	5.45	22.41
	4.	13.41	0.20	1.46	1.88	5.44	22.39
	7.	13.43	0.20	1.48	1.89	5.44	22.44
	10.	13.45	0.21	1.49	1.91	5.46	22.52

*Note:* The reported percentages represent the major identified fatty acids. The remaining fraction consists of unidentified or minor trace fatty acids not presented in this profile.

One of the primary mechanisms affecting fatty acid composition during storage is lipid oxidation, which primarily targets polyunsaturated fatty acids (PUFAs). Studies have shown a significant decrease in PUFA levels during storage, accompanied by an increase in oxidation products such as hexanal and pentanal, known markers of lipid oxidation in various meats (Fortier et al. 2022). Furthermore, saturated fatty acids are less susceptible to oxidation than unsaturated fatty acids (Orkusz et al. 2021).

Research has emphasized that lipid oxidation is a critical factor affecting the quality and safety of stored fish (Jasour et al. 2014). The protective effect of wax coating is to create a barrier against oxygen and moisture, which contribute significantly to oxidation. The addition of essential oil to the coating material enhances this effect.

Wax coating functions both as a barrier and as a facilitator of oxidative conditions. While it protects unsaturated fatty acids, it does not affect the degradation of saturated fatty acids. Therefore, the interaction between lipolytic activity and oxidative stability during storage allows unsaturated fatty acids to remain prevalent or increase in concentration throughout the storage period (Shin et al. 2022).

Wax coatings provide a barrier against external factors against the oxidative degradation of lipids in fish meat. These coatings extend shelf life by minimizing exposure to oxygen and environmental moisture. Wax coatings containing additional essential fatty acids: Essential oils, in addition to the protective effect of beeswax, exhibit greater protective effects against oxidation of fatty acids (especially PUFAs) due to the phenolic substances they contain.

Essential oils derived from plants such as thyme, rosemary, and bay laurel have been found to effectively reduce lipid oxidation and increase the oxidative stability of fish during storage. This protection allows for an increase in the relative concentration of polyunsaturated fatty acids (PUFAs). The observed increase in the unsaturated fatty acid composition of fish meat coated with beeswax containing essential oils during storage is primarily due to the protective antioxidant effects of the essential oils, the wax's ability to protect lipid profiles, and the effects of storage conditions (Gomaa et al. 2019).

Our study results match other research showing that coatings made with chitosan and essential oils can greatly slow down oxidation, which helps keep fish products fresh longer and maintains their fatty acid content (Hashemi et al. 2020).

It was found that the type of fish sample, how long it was stored, and the combination of sample type and storage time (except for lactic acid bacteria count) all had a major impact on the microbiological results of the fish samples. Furthermore, the sample type interaction was negative, while storage time had a significant positive correlative effect on all microbiological analysis results (Table S1).

TAMB, TAPB, and TYM counts in trout samples increased during storage (*p* < 0.05). On the 10th day of storage, the highest TAMB, TAPB, and TYM counts were found in the control sample, with values of 6.20, 5.62, and 6.37 log cfu/g, respectively. Conversely, the lowest TAMB and TAPB counts, with values of 3.21 and 2.91 log cfu/g, were found in the Thyme 4% coded samples, and the lowest TYM count, with a value of 3.95 log cfu/g, was observed in the laurel 4% coded samples.

Similarly, the TCG, LAB, and lipolytic bacteria counts of the samples also increased during storage (*p* < 0.05). The highest increases during storage were 1.17; the control samples showed increases of 2.65 and 2.038 log cfu/g (Table S1).

Although the increase was greatest in the control samples, coating them with beeswax significantly slowed it. Furthermore, the addition of essential oil to the beeswax increased the effect. The resulting effect increased in parallel with the amount of essential fatty acids added.

Casting trout samples with beeswax positively affected their microbiological quality. Furthermore, the addition of essential oil to the coating material positively enhanced this effect. The bioactive compounds with natural antimicrobial properties found in beeswax inhibit the growth of spoilage organisms (Tavares et al. 2021).

Essential oils affect the cell membrane integrity of microorganisms, affecting their metabolic processes and leading to cell death. When added to the wax coating, these essential oils form a protective layer that helps keep the fish fresh and boosts their ability to fight off germs by releasing active substances. Yıldız (2014) reported in his study that thyme essential oil also reduced microbial counts in smoked rainbow trout. He stated that this effect of thyme oil is due to its phenolic compounds, which enhance the oil's preservative properties.

This study aimed to determine the changes in the physicochemical and microbiological quality of trout fillets coated with beeswax and a mixture obtained by adding essential oils (thyme and bay laurel) to the beeswax during storage. It was determined that pH, a_w_, TBA, and TVB‐N values of all samples increased during storage. The beeswax coating significantly reduced these changes, while the addition of essential oil to the coating material further enhanced this effect. Similarly, while the hardness, gumminess, chewiness, resilience L*, and a* values of the stored samples increased, the adhesiveness, springiness, cohesiveness, and b* values decreased. The beeswax coating and the addition of essential oil to the coating material positively affected these changes, with the resulting effect increasing in parallel with the amount of oil added. The beeswax and essential oil mixture wax coatings formed a barrier against oxidative degradation of trout lipids during storage. The resulting antioxidant effect was significantly more effective on PUFAs than on SFAs and MUFAs. Similarly, beeswax coating, and particularly the addition of essential oils to the wax, significantly inhibited microbial growth during storage, helping to preserve the microbial quality of trout fillets.

The beneficial effects of beeswax coatings on physicochemical values, such as TBARS and TVB‐N, enhance food safety and preserve sensory qualities. The inclusion of essential oils in coatings has shown potential in extending the shelf life of seafood by controlling both microbial growth and subsequent biochemical spoilage processes.

The synergistic effects of these natural ingredients suggest a viable approach to preserving fish quality without resorting to synthetic preservatives, making it a promising avenue for future research and application in the seafood industry.

## Author Contributions

**Ayşe Janseli Denizkara:** formal analysis, methodology, validation, visualization, writing – review and editing, writing – original draft. **Gökhan Akarca:** writing – original draft, project administration, supervision, resources, data curation. **Ramazan Şevik:** supervision, project administration, validation, investigation, funding acquisition. **Senem Güner:** conceptualization, methodology, validation, visualization, formal analysis, writing – original draft. **Çiğdem Aşçioğlu:** funding acquisition, writing – original draft, formal analysis.

## Funding

The authors have nothing to report.

## Conflicts of Interest

The authors declare no conflicts of interest.

## Supporting information


**Table S1:** Microbiological analysis results of samples (log cfu/g).

## Data Availability

The data that support the findings of this study are available from the corresponding author upon reasonable request.
