# Supplementary material for: Evaluation of the Effects of Wax Films Formulated With Thyme and Laurel Essential Oils on Chemical and Microbiological Quality Characteristics of Rainbow Trout Fillets During Storage
Source: Food Sci Nutr. 2026 Jun 10;14(6):e71950. doi: 10.1002/fsn3.71950 (PMC13251085; doi:10.1002/fsn3.71950)
Supplement: Supplementary file 1 — Table S1: Microbiological analysis results of samples (log cfu/g). [file FSN3-14-e71950-s001.docx]

| **Analysis** | **Samples** | **Storage Time (Day)** | | | |
| --- | --- | --- | --- | --- | --- |
|  |  | **1.** | **4.** | **7.** | **10.** |
| T**otal Aerobic Mesophilic Bacteria (TAMB) Count** | Control | 3,57±0.02^Da^ | 4,14±0.01^Ca^ | 5,01±0.01^Ba^ | 6,20±0.01^Aa^ |
|  | Beewax | 3,15±0.01^Cb^ | 3,22±0.06^Cb^ | 4,73±0.01^Bb^ | 5,04±0.03^Ab^ |
|  | Thyme %2 | 3,01±0.01^Cc^ | 3,04±0.01^Cc^ | 4,52±0.02^Bc^ | 4,70±0.01^Ac^ |
|  | Thyme %4 | 2,77±0.02^Cd^ | 2,90±0.06^Bd^ | 2,99±0.01^Be^ | 3,21±0.04^Ae^ |
|  | Laurel %2 | 3,01±0.03^Cc^ | 3,01±0.04^Cc^ | 3,17±0.01^Bd^ | 4,08±0.01^Ad^ |
|  | Laurel %4 | 2,69±0.05^Ce^ | 2,85±0.02^Bd^ | 2,93±0.01^ABf^ | 3,01±0.02^Af^ |
|  | **Interactions** | **P Value** | | **r** | |
|  | Sample (S) | <0.0001 | | -0.650** | |
|  | Storage Time (ST) | <0.0001 | | 0.572** | |
|  | S x ST | <0.0001 | | -- | |
| **Total Aerobic Psychrophilic Bacteria (TAPB) Count** | Control | 2,78±0.01^Da^ | 3,31±0.02^Ca^ | 4,11±0.01^Ba^ | 5,62±0.01^Aa^ |
|  | Beewax | 2,74±0.03^Da^ | 3,14±0.03^Cb^ | 4,02±0.01^Ba^ | 5,01±0.01^Ab^ |
|  | Thyme %2 | 2,48±0.03^Db^ | 3,02±0.01^Cb^ | 3,25±0.05^Bb^ | 4,07±0.02^Ac^ |
|  | Thyme %4 | 2,21±0.03^Cc^ | 2,50±0.13^Bc^ | 2,83±0.08^Ac^ | 2,91±0.01^Ad^ |
|  | Laurel %2 | 2,43±0.02^Db^ | 3,01±0.01^Cb^ | 3,18±0.03^Bb^ | 4,01±0.02^Ac^ |
|  | Laurel %4 | 2,05±0.04^Dd^ | 2,28±0.05^Cd^ | 2,56±0.03^Bd^ | 2,78±0.01^Ae^ |
|  | **Interactions** | **P Value** | | **r** | |
|  | Sample (S) | <0.0001 | | -0.562** | |
|  | Storage Time (ST) | <0.0001 | | 0.683** | |
|  | S x ST | <0.0001 | | -- | |
| **Total Yeast/Mold (TYM) Count** | Control | 3,12±0.04^Da^ | 4,05±0.01^Ca^ | 5,01±0.01^Ba^ | 6,37±0.01^Aa^ |
|  | Beewax | 3,01±0.02^Db^ | 3,90±0.01^Cb^ | 4,73±0.01^Bb^ | 5,28±0.02^Ab^ |
|  | Thyme %2 | 2,97±0.03Cb | 3,05±0.02Cc | 3,98±0.04Bc | 5,07±0.04Ac |
|  | Thyme %4 | 2,78±0.05^Dc^ | 2,96±0.04^Cd^ | 4,01±0.01^Bc^ | 4,27±0.01^Ae^ |
|  | Laurel %2 | 2,93±0.03^Db^ | 3,00±0.01^Ccd^ | 3,99±0.01^Bc^ | 4,96±0.01^Ad^ |
|  | Laurel %4 | 2,68±0.01^Dd^ | 2,96±0.04^Cd^ | 3,16±0.03^Bd^ | 3,95±0.04^Af^ |
|  | **Interactions** | **P Value** | | **r** | |
|  | Sample (S) | <0.0001 | | -0.458** | |
|  | Storage Time (ST) | <0.0001 | | 0.815** | |
|  | S x ST | <0.0001 | | -- | |

**TABLE S1** Microbiological analysis results of samples (log cfu/g).

a - f (↓): Values with the same capital letters in the same column for each analysis differ significantly (P< 0.05). A(→)C: Values with the same capital letters in the same column for each analysis differ significantly (P<0.05) *P* < 0.0001: Highly significant. **. Correlation is significant at the 0.01 level (2-tailed). *. Correlation is significant at the 0.05 level (2-tailed).

**TABLE S1** Microbiological analysis results of samples (log cfu/g).

| **Analysis** | **Samples** | **Storage Time (Day)** | | | |
| --- | --- | --- | --- | --- | --- |
|  |  | **1.** | **4.** | **7.** | **10.** |
| **Lactic Acid Bacteria (LAB) Count** | Control | 3,60±0.09^Ca^ | 4,12±0.18^Ba^ | 4,30±0.09^Ba^ | 4,78±0.0^Aa^ |
|  | Beewax | 2,97±0.04^Db^ | 3,52±0.07^Cb^ | 3,78±0.04^Bb^ | 4,34±0.0^Ab^ |
|  | Thyme %2 | 2,34±0.05^Dc^ | 2,77±0.05^Cc^ | 3,30±0.02^Bc^ | 3,74±0.0^Ac^ |
|  | Thyme %4 | 2,06±0.04^De^ | 2,46±0.01^Cd^ | 3,04±0.05^Bd^ | 3,19±0.0^Ad^ |
|  | Laurel %2 | 2,25±0.05^Dd^ | 2,57±0.12^Ccd^ | 3,19±0.01^Bc^ | 3,69±0.0^Ac^ |
|  | Laurel %4 | 1,99±0.04^De^ | 2,32±0.07^Cd^ | 2,89±0.04^Be^ | 3,05±0.0^Ad^ |
|  | **Interactions** | **P Value** | | **r** | |
|  | Sample (S) | <0.0001 | | -0.696** | |
|  | Storage Time (ST) | <0.0001 | | 0.693** | |
|  | S x ST | 0.003 | | -- | |
| **Total Coliform Group Bacteria (TCGB) Count** | Control | 2,60±0.16^Da^ | 3,43±0.02^Ca^ | 4,99±0.04^Ba^ | 5,25±0.01^Aa^ |
|  | Beewax | 2,37±0.08^Dab^ | 3,43±0.02^Ca^ | 3,56±0.01^Bb^ | 4,20±0.02^Ab^ |
|  | Thyme %2 | 2,30±0.04^Dbc^ | 3,25±0.06^Cb^ | 3,37±0.04^Bc^ | 4,05±0.02^Ab^ |
|  | Thyme %4 | 2,21±0.12^Cbc^ | 2,84±0.02^Bc^ | 2,90±0.08^Be^ | 3,59±0.23^Ac^ |
|  | Laurel %2 | 2,24±0.03^Cbc^ | 3,21±0.02^Bb^ | 3,22±0.02^Bd^ | 4,06±0.10^Ab^ |
|  | Laurel %4 | 2,11±0.06^Cc^ | 2,73±0.08^Bc^ | 2,76±0.01^Bf^ | 2,96±0.03^Ad^ |
|  | **Interactions** | **P Value** | | **r** | |
|  | Sample (S) | <0.0001 | | -0.483** | |
|  | Storage Time (ST) | <0.0001 | | 0.783** | |
|  | S x ST | <0.0001 | | -- | |
| **Lipolytic Bacteria Count** | Control | 2,78±0.01^Da^ | 3,43±0.01^Ca^ | 4,01±0.04^Ba^ | 4,82±0.06^Aa^ |
|  | Beewax | 2,60±0.03^Db^ | 3,13±0.03^Cb^ | 3,94±0.07^Ba^ | 4,13±0.03^Ab^ |
|  | Thyme %2 | 2,38±0.03^Dc^ | 3,02±0.01^Cc^ | 3,17±0.01^Bb^ | 3,47±0.04^Ac^ |
|  | Thyme %4 | 1,98±0.06^Ce^ | 2,94±0.05^Bd^ | 3,02±0.01^Bc^ | 3,21±0.02^Ae^ |
|  | Laurel %2 | 2,25±0.02^Dde^ | 3,01±0.02^Cc^ | 3,13±0.03^Bb^ | 3,36±0.01^Ad^ |
|  | Laurel %4 | 1,69±0.06^Cf^ | 2,85±0.02^Be^ | 2,93±0.03^ABd^ | 3,01±0.01^Af^ |
|  | **Interactions** | **P Value** | | **r** | |
|  | Sample (S) | <0.0001 | | -0.541** | |
|  | Storage Time (ST) | <0.0001 | | 0.741** | |
|  | S x ST | <0.0001 | | -- | |

a - f (↓): Values with the same capital letters in the same column for each analysis differ significantly (P< 0.05). A(→)C: Values with the same capital letters in the same column for each analysis differ significantly (P<0.05) *P* < 0.0001: Highly significant. **. Correlation is significant at the 0.01 level (2-tailed). *. Correlation is significant at the 0.05 level (2-tailed).
